# Bacterial colonized melanoma skin models allow to study host–microbe interactions *in situ*

**DOI:** 10.3389/fmicb.2026.1736700

**Published:** 2026-02-24

**Authors:** Aline Rosin, Jannike Lea Krause, Heike Sprenger, Maya Sophie Kissner, Klaus Neuhaus, Tewes Tralau, Tessa Höper, Lisa Lemoine

**Affiliations:** 1 Department of Pesticides Safety, German Federal Institute for Risk Assessment (BfR), Berlin, Germany; 2 Institute for Biotechnology, Technical University of Berlin, Berlin, Germany; 3 Department of Food Safety, German Federal Institute for Risk Assessment (BfR), Berlin, Germany; 4 Core Facility Microbiome, ZIEL–Institute for Food & Health, Technical University Munich, Freising, Germany; 5 German Federal Institute for Risk Assessment (BfR), Berlin, Germany

**Keywords:** 3D skin models, co-cultivation, host–microbe interaction, melanoma, skin microbes

## Abstract

**Introduction:**

Melanoma represents the most lethal form of skin cancer, with the skin microbiome increasingly recognized as a potential risk factor. Previous studies demonstrated an altered microbiome composition at melanoma sites. However, the role of the microbiome remains elusive and technically challenging to investigate. Our proof-of-concept study aims to explore whether the contribution of skin bacteria to melanoma progression can be examined *in situ*.

**Methods:**

We utilized a commercial 3D melanoma model cultivated in an air-liquid interface configuration and apically inoculated it with a diverse bacterial community derived from healthy human skin.

**Results:**

During the 12-day co-cultivation period, bacterial counts were comparable to those found on human skin *in vivo*, with no significant induction of cytotoxicity, although a significant decline in bacterial diversity was observed. Nonetheless, microbial colonization had a clear impact on melanoma biology. This was evidenced by pronounced alterations in gene expression associated with pathways involved in melanoma progression, as well as cadherin switching and increased secretion of cytokines, such as VEGF and GM-CSF, along with the melanoma marker MIA.

**Discussion:**

This study is the first to demonstrate the feasibility of using 3D melanoma models to investigate the impact of skin bacteria on melanoma biology, thereby paving the way for elucidating causal mechanisms *in situ*.

## Introduction

Skin cancer is in the public focus since the increase in UV radiation and exposure thereto became an issue. Despite the increased awareness and closely monitoring of skin cancer, as well as the development of various treatment options through research, the incidence of skin cancer continuously grows, thereby indicating the existence of hitherto unidentified risk factors.

Although melanoma is less common than other types of skin cancer, its incidence rates are rising in Europe over the past five decades (Arnold et al., 2014). Further, the number of new cases worldwide is projected to increase by more than 50% per year between 2020 and 2040 (Arnold et al., 2022). Established risk factors that contribute to its development comprise UV radiation, hereditary markers, and gene mutations (Conforti and Zalaudek, 2021).

Melanoma arises from a malignant transformation of melanocytes, the melanin-producing cells in the skin (Long et al., 2023). This transformation results from the accumulation of gene mutations, leading to apoptosis evasion, uncontrolled cell proliferation, persistent angiogenesis, which lastly allows metastasis formation (Hanahan and Weinberg, 2000). Key changes in melanoma progression include the constitutive activation of the mitogen-activated protein kinase pathway (MAPK) and phosphatidylinositol-3-kinase pathway (PI3K) signaling (Guo et al., 2021). The latter is additionally regulated by the tumor suppressor gene phosphatase and tensin homolog (*PTEN*), which may lose functionality through mechanisms, such as mutation or protein destabilization (Tamguney and Stokoe, 2007). The PI3K/PTEN pathway is also implicated in cadherin switch phenotype, which primes the cell from Cadherin-1 to Cadherin-2 through upregulation of the transcriptional factors Snail and Twist (Hao et al., 2012). These cadherins are involved in an epithelial-mesenchymal transition (EMT)-like switch, where melanoma cells lose cell–cell contact and consequently migrate and invade distant tissues, resulting in metastatic tumor cells (Kong et al., 2011).

To enhance the understanding of melanoma etiology, recent research has shifted focus beyond genetic factors including the skin microbiome (Bouferraa et al., 2023; Routy et al., 2024; L'Orphelin et al., 2025). A potential impact of the skin microbiome on melanoma is currently discussed due to its involvement in cellular processes, such as immune modulation, inflammation, and the maintenance of barrier function (Byrd et al., 2018).

However, only a limited number of studies have investigated the skin microbiome in melanoma. Differential abundance analysis indicated that *Corynebacterium urealyticum* is more prevalent in melanoma samples, whereas the presence of *Roseomonas gilardii* is decreased (Properzi et al., 2025). In patients with advanced stages of acral melanoma, the skin bacterium *Corynebacterium* spp. was significantly more abundant (Mizuhashi et al., 2021). Another study compared melanoma sites with melanocytic nevi and detected a higher number of *Propionibacterium* and *Staphylococcus,* in addition to *Corynebacterium* (Salava et al., 2016). In accordance with this, porcine skin comprised a change in bacterial diversity in melanoma lesions compared to healthy skin (Mrázek et al., 2019). At this stage, these results link the skin microbiome and melanoma progression. However, a detailed characterization of the molecular mechanisms regarding host-microbiome interactions or cause-effect relationships is missing. One reason for this is the lack of suitable model systems for studying such effects *in vivo* or *in situ*.

In this study, we evaluated the suitability of bacteria-inoculated 3D melanoma models for investigating host–microbe interactions within the context of melanoma. We employed the skin cancer model “Melanoma,” onto which we applied a diverse bacterial community obtained from human skin. Following co-cultivation, we examined the effects of bacterial colonization on the melanoma skin models. Analyses comprised cytotoxicity assessments, transcriptomic data of the melanoma models, as well as readouts of several cytokines, melanoma marker, and cadherin switch characterization. A taxonomic overview was generated using 16S rRNA gene amplicon data in addition to cell number counts.

Our study suggests that colonization of melanoma models may serve as valuable tools for further examining the influence of skin microbiota on the progression of melanoma *in situ*.

## Materials and methods

### Chemicals

If not mentioned otherwise, all chemicals were purchased at the highest purity available from Sigma-Aldrich (Taufkirchen, Germany) or Carl Roth (Karlsruhe, Germany).

### Microbial skin tissue co-culture

We utilized Melanoma 3D skin models (MLNM-FT-A375-AFAB) procured from MatTek (Ashland, MA, USA). These were maintained in six-well plates (Techno Plastic Products AG, Trasadingen, Switzerland) containing 2.5 mL of antibiotic-free maintenance medium (MLNM-FT-MM-AFAB) at 37 °C and 5% CO_2_. Following 2 days of recovery with daily medium exchange upon arrival, the models were inoculated with bacteria collected from the volar forearm from a healthy volunteer using sterile omni swabs (QIAGEN GmbH, Hilden, Germany) as described previously (Sowada et al., 2014). The volar forearm exhibits the highest diversity of bacterial species (Grice et al., 2009) and was consequently selected for the collection. The volunteer gave written consent according to German federal government standards. The study has been approved by the Institutional Review Board of the German Federal Institute for Risk Assessment (BfR) with the SFP number 1323–107 and the internal procedural reference number BfR-182.

**Figure 1:**
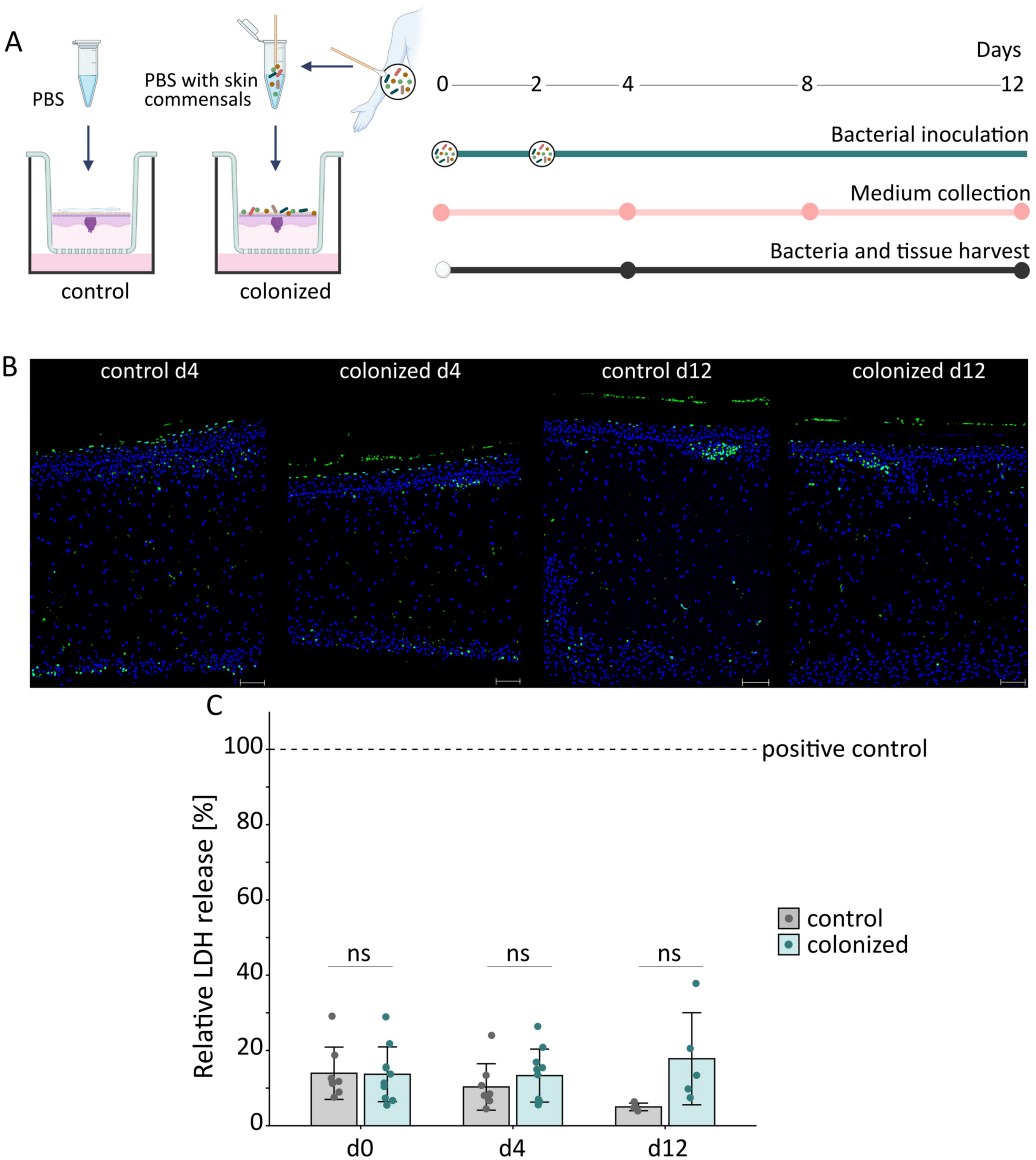
Co-culture of skin bacteria and melanoma models. **(A)** Illustration of the experimental setup. Melanoma models were inoculated twice with skin bacteria extracted from healthy skin sites on day 0 and day 2 and co-cultured for a period of 12 days. Medium was exchanged daily and collected on day 0, 4, 8, and 12. Bacteria and tissues were harvest on day 4 and 12 (Created with BioRender.com). **(B)** Representative images of colonized and non-colonized control melanoma models on day 4 and 12 of co-cultivation. The green fluorescence indicates TUNEL antibody stain, while Hoechst was used to stain the nuclei in blue. Scale bars refer to 100 μm. **(C)** Relative release of lactate dehydrogenase (LDH) from both control and colonized melanoma models normalized to the positive control over the cultivation period. As a positive control, bacteria were introduced into the culture medium of the melanoma model to simulate infection. Data are presented as mean values, with error bars representing ± SD from *n =* 4–10 biological replicates for each condition and time point. Differences between control and colonized melanoma models were analyzed by a multiple unpaired *t*-test with correction for multiple comparison using the Holm–Šidák method (ns: not significant).

Briefly, an area of 1 cm^2^ skin/melanoma model was sampled and the swabs were incubated in 1 mL phosphate-buffered saline (PBS; Ashland, MA, USA) for 10 min at 32 °C and 600 rpm. The swabs were removed using sterile tweezers and the liquid was centrifuged. The cells were resuspended in PBS and 15 μL bacterial suspension were applied on the models, while 15 μL sterile PBS were used for the uncolonized control models. To ensure a successful colonization, melanoma models were colonized twice at intervals of 2 days, i.e., on day 0 and day 2 (Figure 1A).

All melanoma models were cultivated aerobically for a period of 12 days with culture medium being exchanged every day. Culture medium was aliquoted and stored at −80 °C until analyses. The models were harvested either 4 or 12 days post inoculation. Bacteria were removed from the models for quantification and sequencing analysis. Epidermis and dermis were separated using sterile tweezers, and then snap frozen with liquid nitrogen and stored at −80 °C. Melanoma models utilized for tissue sections were transferred into embedding medium (Sakura Finetek, Torrance, CA, USA) and rapidly frozen in liquid nitrogen.

### Bacterial quantification

The quantity of bacteria was determined by taking surface imprints as outlined by Lemoine et al. (2020). In brief, 2 cm^2^ of a sterilized velvet cloth per skin model were soaked in sterile PBS and carefully applied on the surface of the skin models. The velvet was incubated in 1 mL PBS for 30 min at room temperature at 600 rpm to detach the bacteria, wrung out using tweezers and serially diluted to count colony-forming units (CFUs) on Lysogeny Broth (LB) agar plates.

### DNA extraction

Metagenomic DNA was isolated from 350 μL of both skin swabs (inocula), surface imprints from non-colonized control, and colonized melanoma models, as well as DNA extraction reagents to capture the background (sterile PBS, water, and kit control), using the QIAamp UCP DNA Micro Kit (QIAGEN GmbH, Hilden, Germany) following the manufacturer’s instructions. Nucleic acid concentrations were measured using a UV–Vis Spectrophotometer (NanoDrop-ND 1000, Peqlab, Erlangen, Germany) and Qubit (Invitrogen, Carlsbad, CA, USA).

### 16S rRNA gene amplicon sequencing

The V1-V3 regions of the 16S rRNA genes were targeted for amplicon library preparation using primer 27F-YM (5′-AGA GTT TGA TYM TGG CTC AG-3′) and 534R (5′-ATT ACC GCG GCT GCT GG-3′) (Frank et al., 2008; Fierer et al., 2005). Briefly, after a 2-step PCR, which adds adapter and barcodes for sequencing, AMPure XP beads (Beckman Coulter, Krefeld, Germany) were used for amplicon purification. All amplicons were pooled equimolarly and 10% (vol/vol) PhiX standard (Illumina) was added. The final library was sequenced on a MiSeq system using PE300 cartridges v3 with 2 × 275 cycles (Illumina Inc., San Diego, CA, USA) following the manufacturer’s instructions (Reitmeier et al., 2020).

### Data processing of the 16S rRNA gene amplicons

Raw reads of the 16S rRNA gene amplicon data were trimmed 5 bp either side (Haider et al., 2024) and further processed using BiotaWiz, with built-in the Integrated Microbial Next-generation sequencing 2 (IMNGS2) pipeline, an updated version of IMNGS (Lagkouvardos et al., 2016). IMNGS2 is based on UPARSE (Edgar, 2013) and additionally includes the Taxonomy Informed Clustering algorithm for enhanced taxonomic resolution, generating species-like operational taxonomic units (SOTUs) (Kioukis et al., 2022). Briefly, denoised zero-radius OTUs (zOTUs) were clustered within each identified genus to produce SOTUs. Only SOTUs with a relative abundance ≥ 0.25% in at least one sample were kept for further analysis (Reitmeier et al., 2021). Moreover, potential contaminants identified in negative controls and non-inoculated melanoma models were thoroughly removed. SOTU sequences were identified by EzBioCloud’s 16S rRNA gene-based ID (Yoon et al., 2017). Assessment of *α*-diversity based on species richness and taxonomic binning were conducted using Rhea in R version 4.2.3 (Lagkouvardos et al., 2017). For visualization of the taxonomy, taxa with a minimum abundance of 0.5% were retained and plotted using ‘ggplot2’ version 3.5.2 (Wickham, 2016). Non-metric multidimensional-scaling (NMDS) plot of *β*-diversity was calculated by PERMANOVA and visualized using the ‘vegan’ package version 4.4.0 in R (Dixon, 2003).

### Sample preparation for microarray analysis

The extraction of total RNA from the epidermis of the melanoma models was carried out using a TRIzol-based protocol (Chomczynski and Sacchi, 1987). The epidermis was disrupted twice at 25 Hz for 5 min in a TissueLyser II (Qiagen, Hilden, Germany) with TRIzol™ Reagent (Invitrogen, Carlsbad, CA, USA) in accordance with instructions from the manufacturers. The RNA concentration was then measured using a UV–Vis Spectrophotometer (NanoDrop-ND 1000, Peqlab, Erlangen, Germany). RNA-integrity (RIN) was determined on an Agilent 2100 Bioanalyzer System (Agilent Technologies, Waldbronn, Germany) using the Agilent RNA 6000 Nano Kit (Agilent Technologies, Waldbronn, Germany) following the manufacturer’s instructions.

Only RNA samples, which had a RIN > 7 as well as A_260_/A_280_ ratios > 1.5 and A_260_/A_230_ ratios > 1, were used. A minimum of 500 ng RNA per sample were sent for microarray gene analysis. The samples were labeled with a terminal deoxynucleotidyl transferase (TdT) using a proprietary DNA labeling reagent at ATLAS Biolabs (Berlin, Germany), who performed the microarray analysis on Human Clariom S Assays (Applied Biosystems, Foster City, CA, USA).

### Bioinformatic analysis for microarray data

For statistical data analysis and visualization of microarray data, R version 4.4.0 was used (Team RC, 2020). The raw data (CEL files) were normalized and summarized using the robust multiarray average (RMA) algorithm from the R package ‘oligo’ version 1.68.2 (Carvalho and Irizarry, 2010). The genes were associated with vendor-provided annotations and control genes were removed after background correction. Genes with low expression were removed prior to further analysis, leaving 21,250 probes corresponding to 19,358 genes (intensity > 4 in at least two samples). One biological replicate from the non-colonized control melanoma models on day 4 was removed as a likely outlier following quality checks. The remaining data were subjected to differential gene expression analysis using the R package ‘limma’ version 3.60.2 (Ritchie et al., 2015). The linear models were fitted and moderated t-statistics computed using the eBayes function, followed by Benjamini-Hochberg correction for exclusion of false positives. Differentially expressed genes were considered significant if they showed an adjusted *p*-value (adj. p) < 0.05 and a log2 fold change > 0.5 as well as log2 fold change < −0.5. Probabilistic principal component analysis (PCA) was applied on Pareto-scaled and scaled data using the R package ‘pcaMethods’ version 1.96.0 (Stacklies et al., 2007). Volcano plot was displayed using the R package ‘ggplot2’ version 3.5.1.

Gene ontology (GO) term enrichment analysis for biological processes was performed using a hypergeometric test by the R package ‘clusterProfiler’ version 4.12.0 (Wu et al., 2021).

For functional interpretation of differential gene expression results, gene lists were subjected to Ingenuity Pathway Analysis (IPA, version 111,725,566, Qiagen Bioinformatics, Redwood City, CA, USA). Statistical significance was assessed using the integrated Fisher’s exact test, with adjustments made through Benjamini-Hochberg correction.

### qRT-PCR analysis

The reverse transcription of 1 μg RNA was carried out using the high-capacity cDNA reverse transcription kit (Applied Biosystems, Foster City, CA, USA). Reverse transcribed genes were quantified using the fast SYBR Green mix (Applied Biosystems, Foster City, CA, USA) on a QuantStudio 3 Real-Time PCR instrument (Applied Biosystems, Foster City, CA, USA). The specific primers were obtained from Eurofins (Ebersberg, Germany) with the following sequences: *CDH1* forward: 5′-AAG AAG CTG GCT GAC ATG TAC GGA-3′, *CDH1* reverse: 5’-CCA CCA GCA ACG TGA TTT CTG CAT-3′, *CDH2* forward: 5’-CCT CCA GAG TTT ACT GCC ATG AC-3′, *CDH2* reverse: 5′-GTA GGA TCT CCG CCA CTG ATT C-3′, *HPRT* forward: 5′-GTT CTG TGG CCA TCT GCT TAG-3′, *HPRT* reverse: 5’-GCC CAA AGG GAA CTG ATA GTC-3′. *Hypoxanthine–guanine phosphoribosyl transferase* (*HPRT*) was used as reference gene. The relative transcript levels were calculated using the 2^-ΔΔCT^ method (Pfaffl, 2001).

### Detection of released components

Secretion of lactate dehydrogenase (LDH) was assessed using the Cytotoxicity Detection Kit (LDH; Roche, Basel, Switzerland) following the instructions from the manufactures. The absorbance was measured at wavelengths of 490 nm and 690 nm utilizing an Agilent Biotek Synergy 2 plate reader (Thermo Scientific, Schwerte, Germany).

The measurement of cytokine secretion was carried out using a customized LEGENDplex™ multi-analyte bead-based multiplex assay plate according to the instructions of the manufacturer (Biolegend, San Diego, CA, USA) with following analytes: IL-13, IL-8, IL-1a, IL-6, IL-10, IFN-y, VCAM-1, ICAM-1, PIGF, GM-CSF, TIM-3, sRAGE, VEGF. A BD FACS ARIA III (Becton Dickinson, Franklin Lakes, NJ, USA) flow cytometer was used for detection and the LEGENDplex™ data analysis software Suite (Biolegend, San Diego, CA, USA) for data analysis.

The excreted melanoma marker MIA were measured using the ELISA kit for Melanoma Inhibitory Activity Protein 1 (MIA1) (SEH650Hu) from Merck (Darmstadt, Germany) in accordance with the protocols provided by the manufactures.

### Immunofluorescence

Cryopreserved tissues were sectioned, mounted to super frost slides (Thermo Scientific, Waltham, MA, USA) and fixed in ice-cold methanol. After evaporization, the tissues were rehydrated in DPBS (Pan-Biotech, Aidenbach, Germany). The cryosections were permeabilized in 0.5% Triton X-100 in DPBS for 9 min, washed twice with DPBS, and blocked with 0.2% Tween and 3% bovine serum albumin (BSA) in DPBS for 40 min. Slides were incubated with primary antibody for 2 h, washed, and incubated with secondary antibody for 50 min. The following antibodies were applied and diluted in blocking solution: Cadherin-1 1:30 (33–4,000; Thermo Scientific, Waltham, MA, USA), Cadherin-2 1:30 (18–203; Abcam, Cambridge, UK), Alexa fluor 488 Goat anti-Rabbit IgG (H + L) 1:200 (A-11008, Thermo Scientific, Waltham, MA, USA) and Alexa fluor 488 Goat anti-Mouse IgG (H + L) 1:400 (A-11001, Thermo Scientific, Waltham, MA, USA).

After antibody staining, slides were washed three times using 0.5% Triton X-100 in DPBS and then once in DPBS. Sections were counterstained with 1 μg/mL Hoechst in DPBS for 10 min and mounted in Fluor Save Reagent (Sigma-Aldrich, Taufkirchen, Germany). Stained slides were examined using a LSM700 confocal microscope (Carl Zeiss, Oberkochen, Germany) with the “Tile Scan” function in the ZEN 2012 black edition software (Carl Zeiss, Oberkochen, Germany), using a 2×2 tile configuration to create composite images. Relative fluorescence intensity was analyzed using the ZEN 3.1 lite blue edition software (Carl Zeiss, Oberkochen, Germany). The whole image was marked as region of interest, for which the fluorescence intensities were extracted.

To assess cell apoptosis, tissue sections were stained with the TUNEL *In Situ* Cell Death Detection Kit, Fluorescein kit (Roche, Basel, Switzerland), following the manufacturer’s instructions. The stained tissues were then analyzed using the fluorescent microscope BZ-X (KEYENCE, Neu-Isenburg, Germany).

### Data visualization and statistics

Unless otherwise specified, data visualization was conducted using the R package ‘ggplot2’ version 3.5.2, and heatmaps with clustered dendrograms were generated with ‘pheatmap’ version 1.0.13 within the R environment version 4.4.3.

Statistical tests were conducted using R programming or GraphPad Prism version 10.1.2 (Graph Pad, La Jolla, CA, USA) with significance levels of *p < 0.05, **p < 0.01, ***p < 0.001, and ****p < 0.0001. The specific details of each statistical analysis can be found in the corresponding method descriptions and figure legends.

The experimental setup graphic and the summarizing figure were graphically illustrated using BioRender.

## Results

Comprehensive insights into the role of the human skin microbiome on melanoma development remain elusive due to limitations in available experimental systems. Currently, animals reflect the gold standard although they possess limited transferability to human. This arises from the substantial differences between humans and animals, particularly in terms of skin composition (Brettmann and de Guzman, 2018), metabolism (Oesch et al., 2014), and immune responses (Pasparakis et al., 2014). Those factors influence both host-microbe and microbe-microbe interactions.

Thus, we investigated the feasibility of studying human host–microbe interactions in melanoma progression *in situ*. The melanoma models comprised primary epidermal keratinocytes and dermal fibroblasts, and cells from the malignant melanoma cell line A375.

### Prolonged co-cultivation reduces bacterial diversity without affecting skin cell viability

To ensure successful colonization with bacteria, melanoma models were inoculated with skin bacteria twice at intervals of 2 days, i.e., on day 0 and day 2 (Figure 1A). The melanoma models were cultivated for 12 days, with scheduled measurements of cytotoxicity and bacterial growth on days 4 and 12 after bacterial inoculation. During the entire cultivation period, neither the control nor the colonized melanoma models exhibited significant reduction in cell viability, as evidenced by the absence of DNA fragmentation in the TUNEL assay (Figure 1B) and consistent LDH release (Figure 1C).

**Figure 2:**
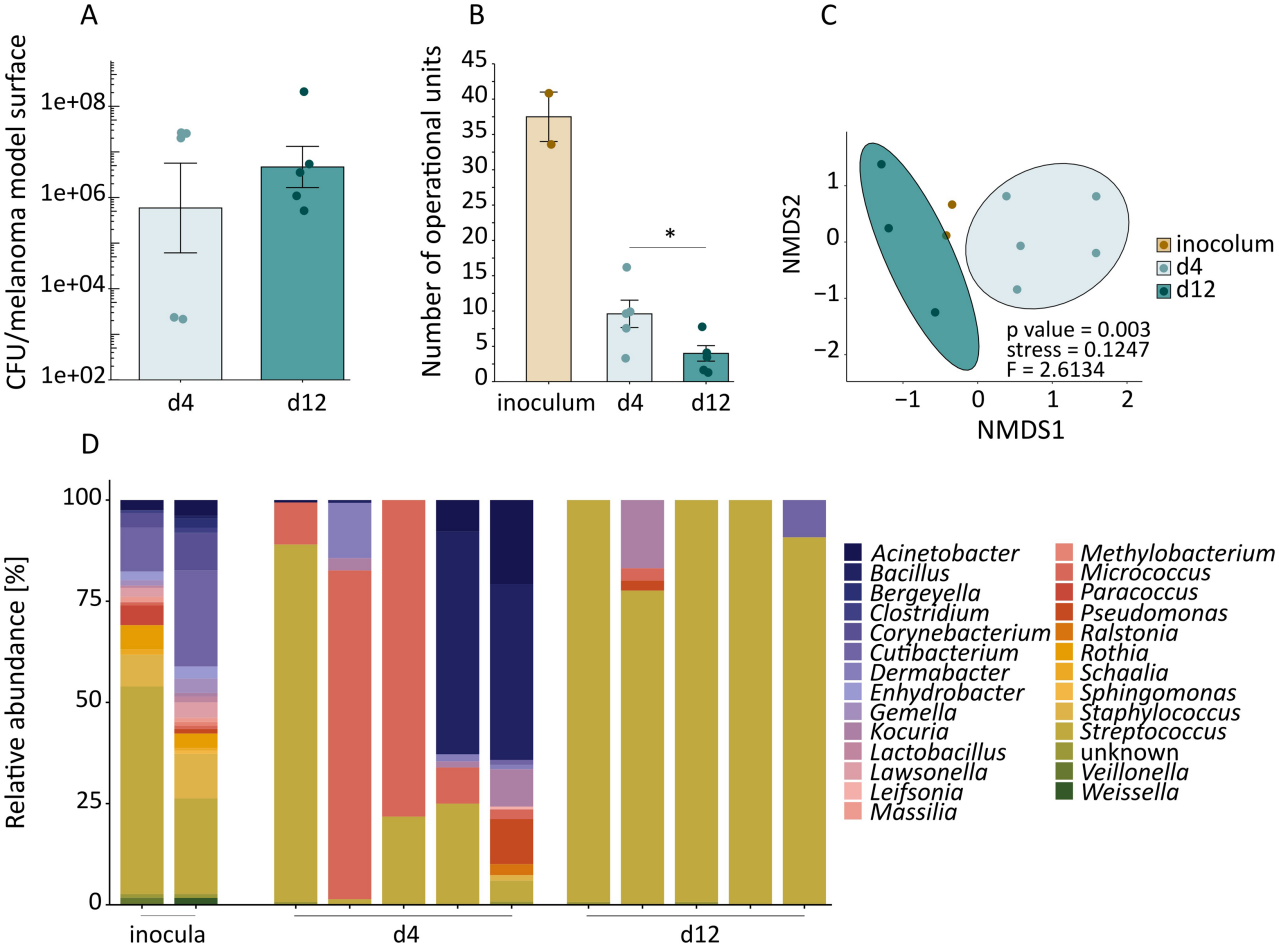
Analyses of skin bacteria used for inoculation and collected from melanoma models after co-cultivation. **(A)** Colony forming units (CFUs) of the bacterial culture from surface imprints of melanoma models on day 4 and 12 post-bacterial inoculation (mean with ± SD from *n =* 5 biological replicates for each time point). 16S rRNA gene amplicon sequencing of bacterial communities from two inocula (*n =* 2) used for inoculation of melanoma models as well as bacterial communities collected from colonized melanoma models on day 4 and 12 (*n =* 5 biological replicates for each time point) shown as **(B)** *α*-diversity indicated as effective richness mean with ± SD, difference between day 4 and day 12 was analyzed by two-tailed unpaired *t*-test (*p < 0.05) and **(C)** β-diversity shown as a non-metric multidimensional-scaling (NMDS) plot. **(D)** Relative abundance of bacterial populations from two inocula used for inoculation and colonized melanoma models on day 4 and 12.

The colonized melanoma models exhibited viable colonization, with mean counts of 1 × 10^6^ CFUs on day 4 and 1 × 10^7^ CFUs on day 12 per skin model (Figure 2A). Concomitantly, microbial composition was monitored by 16S rRNA gene amplicon sequencing. With respect to the inocula, the *α*-diversity was clearly reduced on day 4 and showed a further decline by day 12 (Figure 2B). The taxonomic composition of the skin swabs was initially similar, but changed during cultivation by day 4 and day 12 (Figure 2C). Both skin swabs exhibited a diverse microbiota, encompassing at least 26 distinct genera (Figure 2D). Despite the high α-diversity, the day 0 inoculum comprised ∼50% *Streptococcus*, whereas the day 2 inoculum contained 25% *Streptococcus*. The abundance and composition changed during co-cultivation (Figure 2D). On day 4, the bacterial composition varied among replicates. The predominant genera in the high CFUs melanoma models were either *Streptococcus* (Replicate 1) or *Micrococcus* (Replicate 2 and 3), while the two melanoma models with low CFUs (Replicate 4 and 5) were comparably diverse with a significant presence of *Bacillus*. On day 12, bacterial composition was consistent across replicates, with *Streptococcus* as predominant genus, likely attributable to culture-effects.

### Colonizing melanoma models affects skin model biology

To characterize the influence of the skin bacteria on the physiology of the melanoma models, the transcriptomic responses of the epidermis and cytokine release were analyzed.

#### Significant alterations in the transcriptome of colonized skin models

Three replicates of colonized melanoma models with a bacterial cell number of ∼1 × 10^7^/skin model on day 4 and ∼1 × 10^6^/skin model on day 12 were utilized for the gene expression analysis. The assay interrogates 20,000 well-annotated human genes and thus provides valuable insights into tissue physiology and biological response priming. Similarities of gene expression profiles related to melanoma model conditions were visualized using PCA (Supplementary Figure S1). On day 4, the control and colonized models exhibited high variability among replicates. Conversely, by day 12 replicates were more consistently grouped according their respective conditions, demonstrating reduced variability.

**Figure 3:**
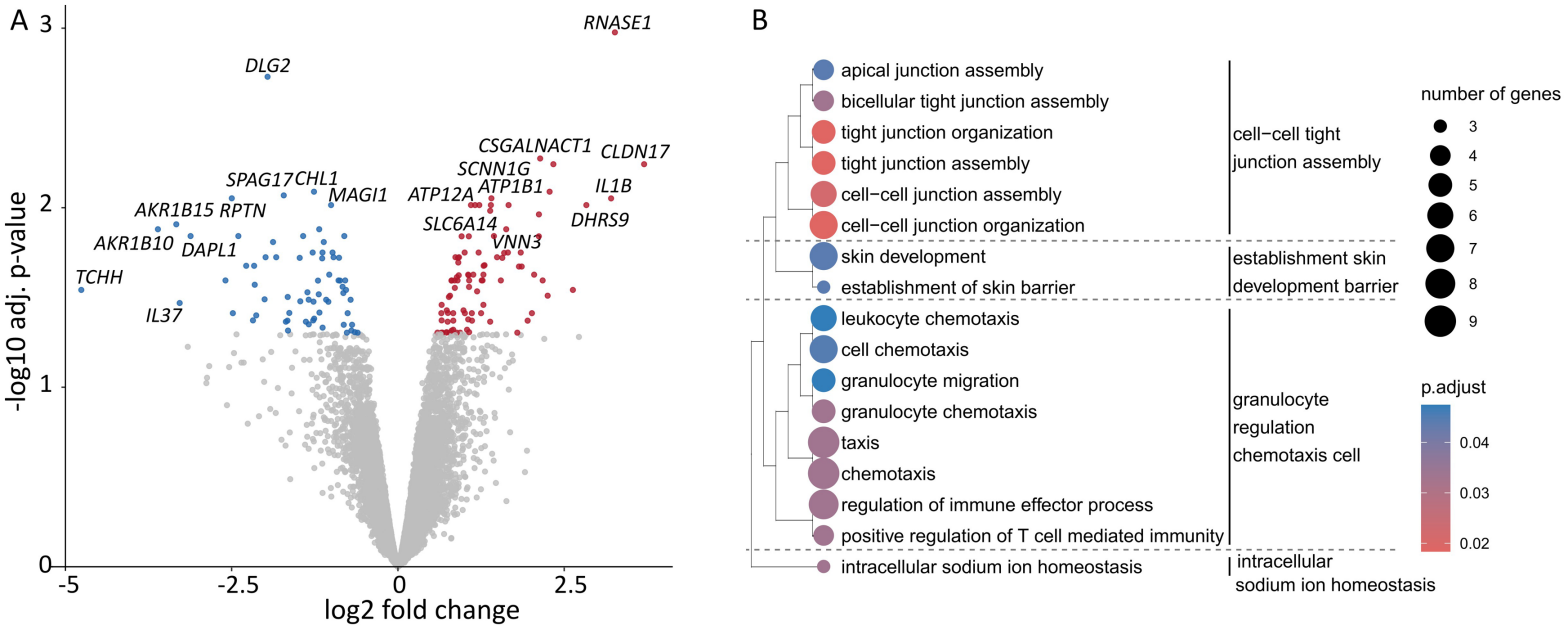
Epidermal transcriptome analysis of colonized melanoma models on day 12. **(A)** Volcano plot depicting log2 fold change and -log10 adjusted *p*-value (adj. *p*). The data indicates differentially expressed genes normalized to non-colonized control melanoma models from *n =* 3 biological replicates for each condition (adj. *p <* 0.05; down: log2 fold change < 0.5, blue; up: log2 fold change > 0.5, red as calculated by *t*-statistics with eBayes function and Benjamini-Hochberg correction). **(B)** Dot-plot of gene ontology (GO) enrichment analysis for biological processes based on significantly differentially upregulated genes showing induced pathways in colonized melanoma models on day 12 of cultivation, normalized to non-colonized control from *n =* 3 biological replicates for each condition. Size and color of the dots represent number of genes per functional group and adj. *p* significance level, respectively.

Marked transcriptional changes related to microbial colonization were primarily observed at the later stages of co-colonization, that is on day 12. Overall, 153 differentially expressed genes (DEGs) were detected, of which 86 were upregulated. Significantly upregulated genes comprised *SCNN1G, SLC6A14, CSGALNACT1, VNN3, ATP1B1, ATP12A*, *IL1B, DEFB4A,* and *DEFB4B* (Figure 3A). Pathway enrichment analysis (≥ 3 DEG-matches for a given GO) of the upregulated genes indicated functional priming toward extracellular matrix remodeling, including cell–cell tight junction assembly, immune modulation, and changed sodium homeostasis (Figure 3B), whereas downregulated transcripts were almost exclusively associated with metabolic processes (Supplementary Figure S2).

#### Microbial colonization impacts release of various cytokines

**Figure 4:**
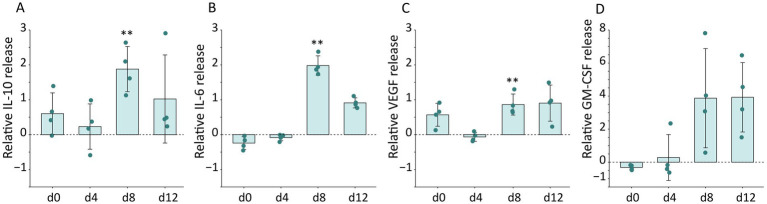
Release of cytokines of colonized melanoma models normalized to non-colonized control melanoma models. Protein levels of **(A)** IL-10, **(B)** IL-6, **(C)** Vascular endothelial growth factor (VEGF), and **(D)** Granulocyte macrophage colony-stimulating factor (GM-CSF) secreted in the cell culture supernatant over the cultivation period of 12 days. Measurement was performed using flow cytometry-based multiplex immunoassay. Data are presented as mean with ± SD of fold changes relative to control melanoma models, with a subtraction of one, from *n =* 4 biological replicates for each time point and condition. Differences between control and colonized melanoma models were analyzed by a multiple unpaired *t*-test with correction for multiple comparison using the Holm–Šidák method (**p < 0.01).

To investigate an immunogenic response, predominantly mediated by keratinocytes given the absence of immune cells, secreted cytokines were quantified (Figure 4; Supplementary Figure S3). The anti-inflammatory cytokine IL-10 was significantly elevated in colonized melanoma models on day 8 compared to the non-colonized melanoma models, showing a slight decrease by day 12 (Figure 4A). This was also observed for IL-6, which may possess both pro-inflammatory and anti-inflammatory properties depending on the physiological context (Figure 4B). The reduction of both interleukins on day 12 may suggest a potential adaptation to the applied bacteria. Furthermore, microbial colonization was found to initiate the release of vascular endothelial growth factor (VEGF; Figure 4C), a critical factor for angiogenesis, as well as granulocyte macrophage colony-stimulating factor (GM-CSF; Figure 4D), which plays a role in the differentiation of immune cells. Notably, those cytokines did not exhibit a reduction by day 12.

Biologically, the transcriptomic responses of the colonized melanoma models shared features with the physiology expected in a cancer model. In addition, bacterial surface counts corresponded to those on human skin *in vivo* (Ross et al., 2019) and no indication of acute skin defense responses such as antimicrobial peptides (AMPs) was observed in the transcriptome analysis.

### Bacterial colonization impacts melanoma associated pathways and markers

**Figure 5:**
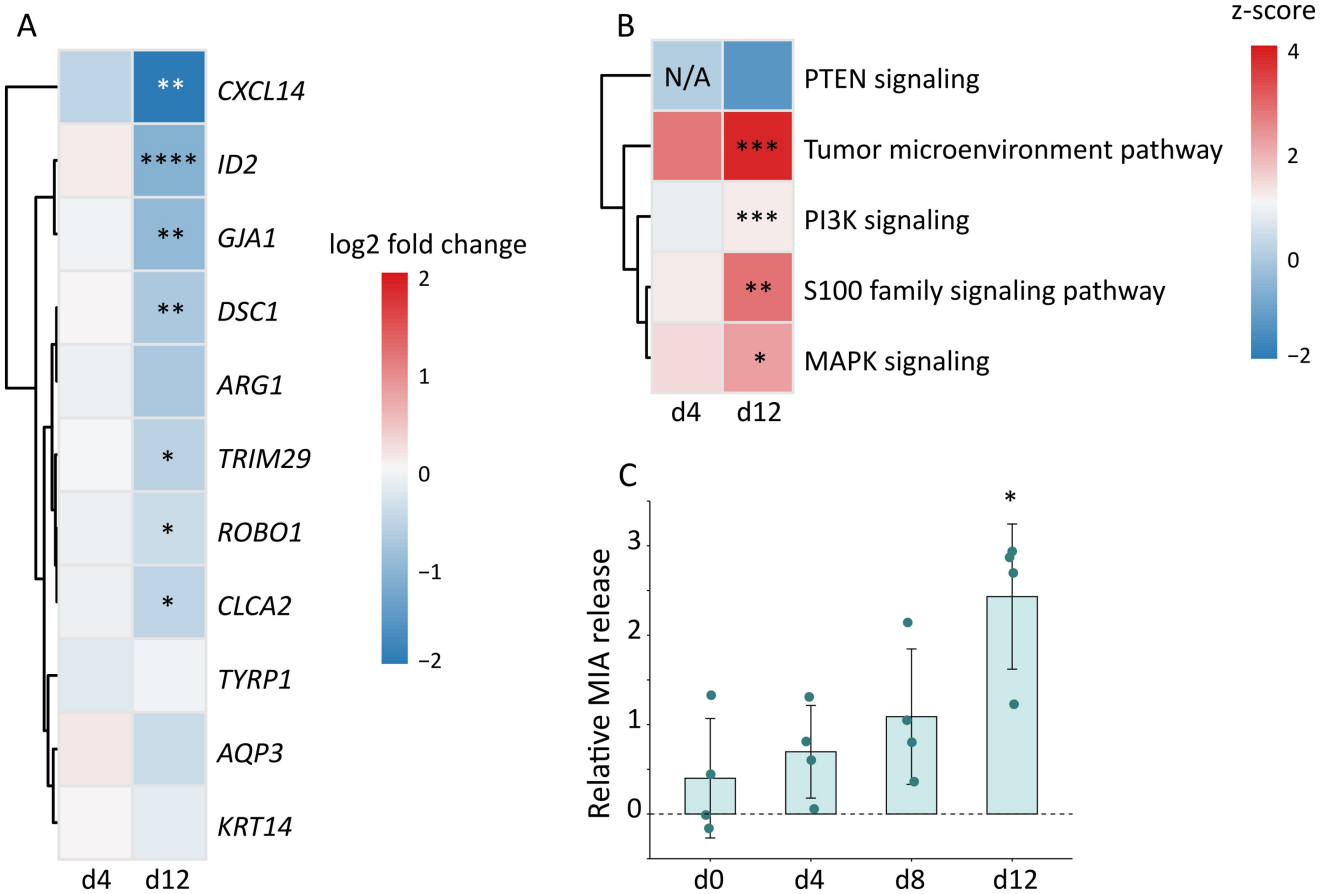
Expression of melanoma markers and melanoma-associated pathways in colonized melanoma models normalized to non-colonized control melanoma models. **(A)** Expression profile of genes involved in metastasis progression in colonized melanoma models. Values indicate log2 fold change on day 4 and 12 from *n =* 3 biological replicates for each condition and time point; except from non-colonized control melanoma models on day 4 with *n =* 2 biological replicates. Significance was analyzed by *t*-statistics with eBayes function and Benjamini-Hochberg correction and displayed as adjusted *p*-values (*adj. *p <* 0.05, **adj. *p <* 0.01, ****adj. *p <* 0.0001). **(B)** Differentially expressed melanoma-associated pathways based on z-scores on day 4 and 12 from *n =* 3 biological replicates for each condition and time point; except from non-colonized control melanoma models on day 4 with *n =* 2 biological replicates. Significance was analyzed by Fisher’s exact test and Benjamini-Hochberg correction and displayed as adjusted p-values (*adj. *p <* 0.05, **adj. *p <* 0.01, ***adj. *p <* 0.001). **(C)** Released levels of melanoma-derived growth regulatory protein (MIA) in the cell culture supernatant measured on day 0, 4, 8, and 12 using ELISA. Data are presented as mean values with ± SD from *n =* 4 biological replicates for each time point and condition. Differences between control and colonized melanoma models were analyzed by a multiple unpaired t-test with correction for multiple comparison using the Holm-Šidák method (*p < 0.05).

For a deeper understanding of the microbial contribution to melanoma biology, expression of selected melanoma markers was assessed and compared to the gene signature for metastatic potential of melanoma proposed by Gerami et al. (2015). According to this study, the metastatic signature mainly relies on the downregulation of 24 relevant genes. Of these 24 genes, 11 transcripts were downregulated in our melanoma models upon colonization (Figure 5A). Of those, *ID2, CXCL14, DSC1, GJA1, ROBO1, TRIM29*, and *CLCA2* were significantly downregulated on day 12, suggesting that the regulation toward metastasis development was more pronounced by day 12.

Transcriptome data were further analyzed using Ingenuity Pathway Analysis (IPA), which identifies biological processes from a given data set, including biological pathways, gene networks, and disease mechanisms. IPA revealed an induction of melanoma-associated pathways in colonized epidermis on day 4 and 12 (Figure 5B). Interestingly, genes from the MAPK and PI3K signaling pathways relevant for melanoma progression, were significantly higher expressed in the colonized melanoma models. Furthermore, genes related to tumor microenvironment and S100 family signaling, which both impact the invasion and survival of tumor cells, were also significantly increased. In contrast, PTEN signaling was downregulated, which is consistent with PI3K pathway induction, as PTEN negatively regulates PI3K (Tamguney and Stokoe, 2007).

In line with this, the melanoma-derived growth regulatory protein (MIA) was detected at increasing levels over the course of cultivation, reaching significance on day 12 (Figure 5C).

### Cadherin switch from Cadherin-1 to Cadherin-2 in colonized melanoma models

**Figure 6:**
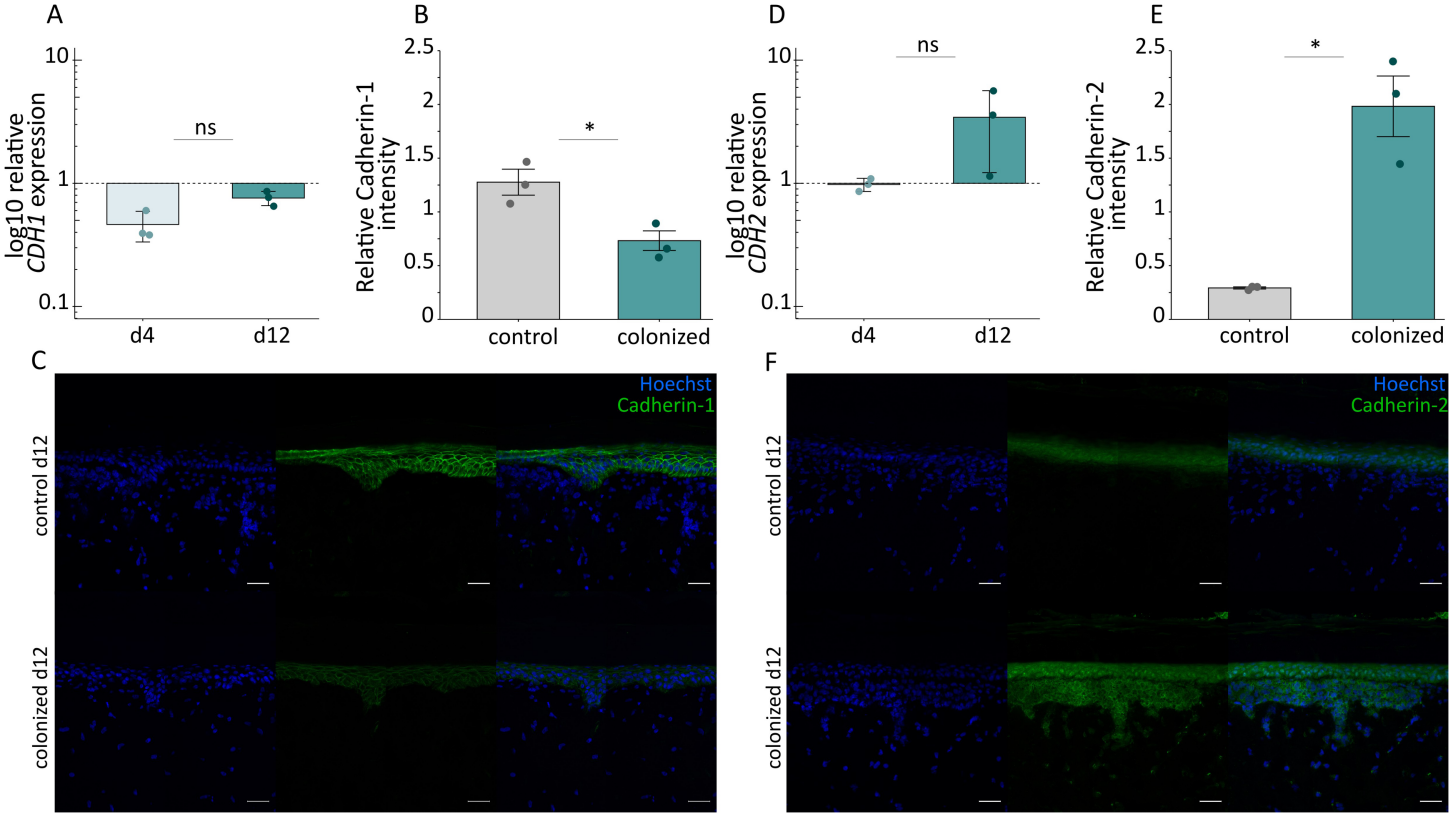
Cadherin switch in colonized and control melanoma models. Relative gene expression of **(A)** *CDH1* and **(D)** *CDH2* in the epidermis of colonized melanoma skin tissues on day 4 and 12 as measured by qRT-PCR. Shown are mean values with ± SD recorded in tissue from colonized models and normalized to non-colonized control from *n =* 3 biological replicates for each condition and time point. Relative intensities of **(B)** Cadherin-1 and **(E)** Cadherin-2 normalized to Hoechst intensities of colonized and non-colonized control melanoma models on day 12 from *n =* 3 technical replicates for each condition. Representative images of skin tissues from colonized or non-colonized control models on day 12, immunostained against **(C)** Cadherin-1 or **(F)** Cadherin-2. The green fluorescence indicates antibody stain, while Hoechst was used to stain the nuclei in blue. Images were captured using the “Tile Scan” function of the Carl Zeiss LSM700 confocal microscope. Scale bars refer to 50 μm. Differences between non-colonized control and colonized melanoma models were analyzed by two-tailed unpaired *t*-test (*p < 0.05, ns: not significant).

As the GO term enrichment analysis showed that cell–cell tight junction assembly was affected in the colonized melanoma models (Figure 3B), we quantified the expression of Cadherin-1 and Cadherin-2, which both play a role in cell adhesion. Therefore, we examined mRNA levels of the corresponding genes and visualized both proteins by immunofluorescent staining (Figure 6).

The relative expression of *CDH1* was clearly extenuated upon colonization (Figure 6A). In concordance with this, intensities of protein levels of Cadherin-1 were significantly downregulated (Figures 6B,C). Concomitantly, the relative expression of *CDH2* was upregulated on day 12 after bacterial inoculation (Figure 6D) with protein staining revealing a significant increase for Cadherin-2 (Figure 6E), which extended into the dermal skin layers in the colonized melanoma models (Figure 6F).

Overall, the data presented here indicate a strong impact of bacterial colonization on melanoma biology. Transcriptomic changes translate, among others, into enhanced expression and excretion of metastasis markers and a switch from Cadherin-1 to Cadherin-2.

## Discussion

**Figure 7:**
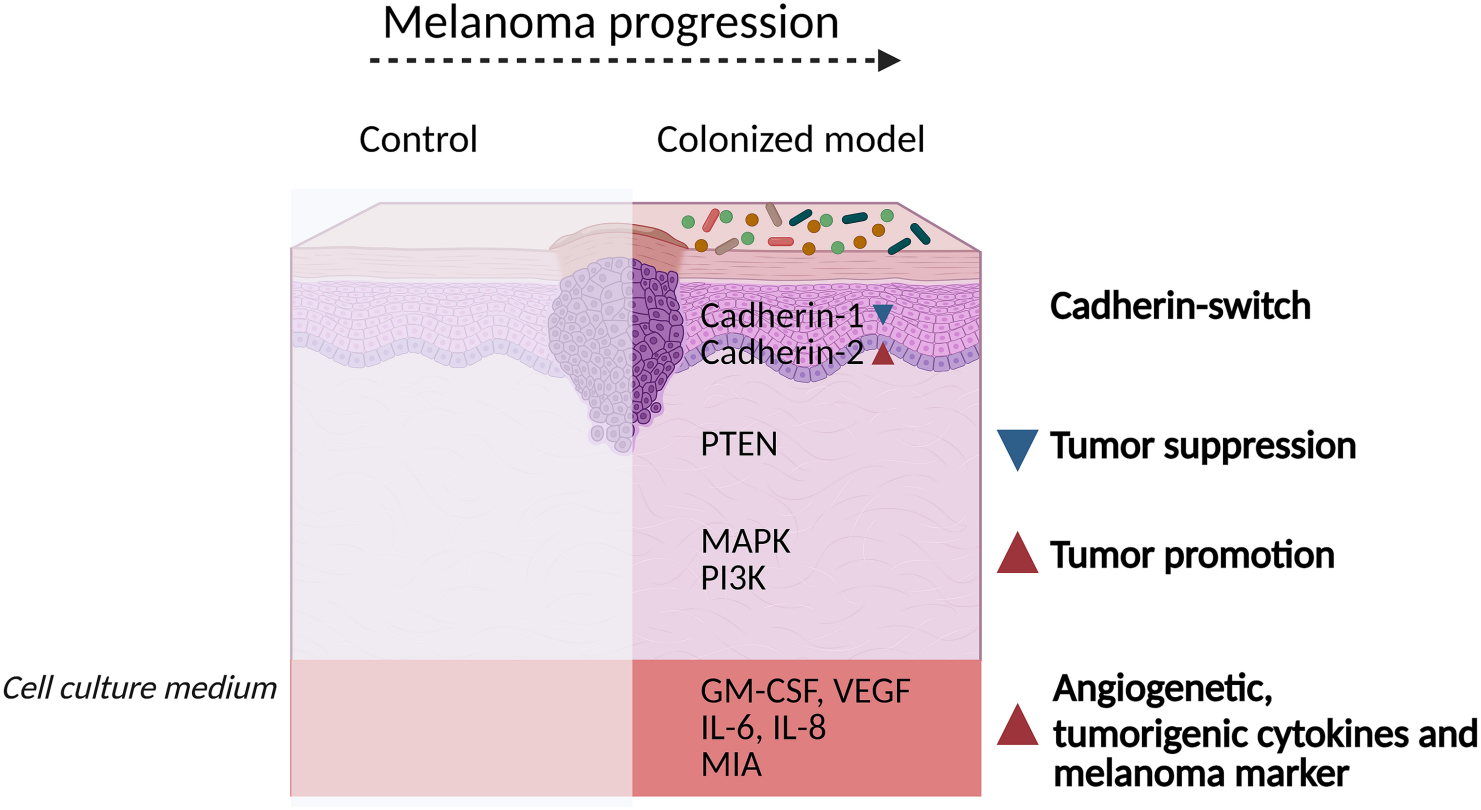
Summary figure describing key findings in bacterial colonized melanoma models. Colonization induced a cadherin-switch characterized by downregulation of Cadherin-1 and upregulation of Cadherin-2. Moreover, the tumor suppressor signaling pathway PTEN was attenuated, while tumor-promoting pathways, such as MAPK and PI3K were upregulated. The secretion of angiogenetic cytokines GM-CSF and VEGF, as well as tumorigenic interleukins IL-6 and IL-8, along with the melanoma marker MIA, were enhanced. Overall, colonized melanoma models indicate an acceleration in melanoma progression.

Data on melanoma and the role of the skin microbiome predominantly comes from studies using mouse models (Nakatsuji et al., 2018; Chen et al., 2023). While these unarguably possess all the advantages of a systemic model, they are subject to inherent limitations due to inter-species differences in physiology and metabolism, or the representativeness of the murine microbiome compared to human skin (Gerber et al., 2014). For a better characterization of the role of the microbiome in melanoma inhibition, promotion, and progression, models closer to the human biology are required. As a proof of concept, we investigated whether microbiome harboring 3D melanoma skin models can be effectively utilized to investigate human host–microbe interactions. The colonized melanoma models remained viable throughout the cultivation period and key events associated with melanoma progression were influenced (Figure 7).

During co-cultivation bacterial composition shifted, which was accompanied by a reduced bacterial diversity and a notable prevalence of *Streptococcus* sp. Alterations in microbial composition upon and during *in vitro* cultivation are common and depend on multiple factors, including nutrient composition (Średnicka et al., 2023) or pH (Haindl et al., 2021). A recent study using an *ex vivo* porcine skin model has also demonstrated an alteration in the skin microbiome, characterized by a reduction in taxonomic diversity and a predominance of *Staphylococcus* (Townsend et al., 2025). The observed decrease in diversity is likely attributable to cultivation and potentially nutrient depletion. Moreover, the high abundance of *Streptococcus* sp. in the inocula provided this genus with a growth advantage, potentially contributing to its dominance on day 12. Nevertheless, *Streptococcus* sp. has been previously identified in skin cancer among the most abundant genera in melanoma tissues from pigs (Mekadim et al., 2022), but also as significantly increased in basal cell carcinoma (Madhusudhan et al., 2020).

Alterations in bacterial composition are not only influenced by niche and nutrient availability (Qiao et al., 2024), but also by host factors (Byrd et al., 2018), such as the secretion of AMPs. These peptides are primarily produced by keratinocytes in response to microbial stimuli (Zheng et al., 2020). They protect epithelial sites from pathogen invasion and shape the indigenous microbial communities in regard to number, composition, and distribution (Gallo and Hooper, 2012). In the colonized melanoma models, gene expressions of the common AMPs *DEFB4A* and *DEFB4B* have been upregulated, reflecting an active response of the skin toward the microbial population. This is in line with previous findings from our group (Lemoine et al., 2020) that showed that commensal bacteria trigger DEFB4A secretion in a similar *in situ* skin model. This supports the notion that the microbiome influences AMP synthesis, which in turn is essential for skin homeostasis by microbial growth restriction. In addition, AMPs may contribute to skin diseases (Niyonsaba et al., 2017) and mediate malignancy in melanoma (Jia et al., 2017; Kim et al., 2010), which should be a focus in future research in microbiome-melanoma-interactions. In addition to AMPs, host derived immune mechanisms, which are mediated among others by cytokines, regulate the skin microbiota (Lunjani et al., 2021) and vice versa (Narros-Fernández et al., 2024). In the colonized melanoma models, the gene expression of *IL1B* and the levels of inflammatory cytokines IL-6 and IL-8 have significantly been elevated. Due to the lack of immune cells the effects are merely suggestive and may demonstrate the direct cytokine response of keratinocytes to the skin microbiome. Keratinocytes possess pattern recognition receptors (PRRs) that identify the microbiome and are capable of cytokine production, thereby mediating immune tolerance and protecting the skin barrier from pathogens (Jiang et al., 2020). Commensals induce low secretion of IL-1B in human keratinocytes, while disease-associated microbes fuel IL-1B release (Narros-Fernández et al., 2024). Alike, the synthesis of IL-6 and IL-8 in dermal cells may result from microbial stimulation via PPRs and subsequent NF-κB signaling (Juráňová et al., 2018). For instance, *Escherichia coli* upregulates the expression of IL-8 in colorectal cancer (Cane et al., 2010). The simultaneous and high release of IL-10 in our models indicates immune tolerance to the microbiome, since no signaling pathways directly linked to antimicrobial defense were activated (Fung et al., 2016). The aforementioned cytokines are associated with increased tumorigenic potential in melanoma (Itakura et al., 2011; Filimon et al., 2021; Hoejberg et al., 2012), which is particularly true for IL-6 and IL-8 that may activate MAPK signaling (Meier and Brieger, 2025; Zhao et al., 2020).

Hyperactivated MAPK signaling is central in melanoma and in the pathogenesis of various other cancer types (Braicu et al., 2019). MAPK can also directly be activated by bacterial virulence factors, such as the cysteine protease gingipain from *Porphyromonas gingivalis*, triggering the promotion of colorectal cancer (Mu et al., 2020). Furthermore, the skin microbe *Staphylococcus aureus* has been shown to activate MAPK via TLR2 signaling (Menzies and Kenoyer, 2006). Similarly, *Streptococcus* sp., which was dominant in our co-culture, can activate MAPK signaling in keratinocytes via M1-protein–dependent mechanisms, promoting inflammation (Persson et al., 2015). Besides MAPK, PI3K represents a core tumorigenic pathway, which is repressed by PTEN. Microbial induction of PTEN has been associated with lung cancer (Tsay et al., 2018).

In general, tumor signaling pathways can be influenced by bacterial products through a variety of mechanisms (Zhou et al., 2022), with secreted components playing a key role in bacteria-host crosstalk (Sanam et al., 2025). A recent study demonstrated that secreted lipids by *S. aureus* generated by the lipase Sal2 promotes melanoma clustering and invasion in zebrafish (Giese et al., 2024). Bacterial metabolites not only directly influence tumor progression, they also modify the tumor microenvironment and inflammatory mediators (Rossi et al., 2020). VEGF is a prevalent inflammatory mediator overexpressed in cancer (Mabeta and Steenkamp, 2022). It is involved in stimulating angiogenesis, a process in which new blood vessels are formed to supply tumor sites with nutrients and oxygen (Mahabeleshwar and Byzova, 2007) and prerequisite of metastasis formation. Excretion of VEGF was increased following microbial colonization of our melanoma models. In line with this, modulation of VEGF expression has been demonstrated for several bacteria, such as *E. coli*, *Bacteroides fragilis* or *Enterococcus faecalis* (Wang et al., 2024). Another growth factor that was induced in our colonized melanoma models was GM-CSF, which may be produced by melanoma cells (García-Martínez et al., 2025). GM-CSF has immunomodulatory functions and has proven a dual role in cancer immune mechanisms being either stimulatory or suppressive (Hamilton, 2015). Constitutive expression of GM-CSF was previously detected in skin carcinogenesis (Mueller et al., 2001). Moreover, GM-CSF is an inducer of angiogenesis and as such a potential promoter for another key feature for the survival and progression of tumorigenic tissue (Zhao et al., 2014). Potential modifications within the tumor microenvironment should be regarded as only indicative, given that the melanoma models employed are not immune-competent.

A further involvement of colonization in melanoma progression was evidenced by the upregulation of *CSGALNACT1*, *SCNN1G*, and *SLC6A14* gene expression, which are also elevated in melanoma cells (Bychkov et al., 2021) and in fibrolamellar carcinoma, a type of liver cancer (Francisco et al., 2022). The amino acid transporter *SLC6A14* is a tumor promoter in colon (Sikder et al., 2020) and pancreatic cancer (Dang et al., 2024). Further, the genes *ATP1B1* and *ATP12A*, which encode an ATPase proton pump and regulate intracellular sodium ion homeostasis, were upregulated upon colonization. This in turn is crucial for the regulation of glycolysis and thus cellular energy levels (Michaels et al., 2024). Malignant cells primarily rely on glycolysis for proliferation, even in the presence of abundant oxygen, a phenomenon known as the Warburg effect (Warburg et al., 1927). Moreover, we observed transcriptional activation of pathways involved in cell–cell tight junction assembly and changes in the expression of key proteins of the adherens junctions Cadherin-1 and Cadherin-2. Downregulation of Cadherin-1 and upregulation of Cadherin-2 are indicative of an EMT-like switch, which is critical in the formation of metastasis in melanoma. In more detail, Cadherin-1 mediates cell–cell contact of keratinocytes with melanocytes, whereas Cadherin-2 mediates interactions of melanocytes with melanocytes, as well as with fibroblasts (Haass et al., 2004). As a result, melanocytes detach from their cell–cell connections and may invade distinct tissues. The degradation of Cadherin-1 is influenced by bacterial proteases, including HtrA protease from *Helicobacter pylori* (Hoy et al., 2012), fragilysin from *B. fragilis* (Wu et al., 1998), as well as the above mentioned gingipains from *P. gingivalis* (Katz et al., 2002). The EMT-like switch may also be promoted indirectly by bacterial-induced inflammation via the action of pro-inflammatory cytokines (Gupta et al., 2022). Both mechanisms are plausible, suggesting progression of melanoma toward a metastatic phenotype. This is supported by the increased secretion of the protein MIA, a prognostic marker for metastatic melanoma states (Sandru et al., 2014).

At this stage, our study does not distinguish between a sustained activation of tumor-promoting signaling pathways or a short-term stress response. Microbial colonization can activate tumor signaling pathway, such as MAPK, during acute host response (Kirk et al., 2020), but microbial-related barrier damage or alterations in microbial composition can promote chronic inflammation and immune dysregulation, which facilitates tumor initiation and progression (Singh et al., 2025; Liu et al., 2025). The reduction in species diversity observed during cultivation could indicate the onset of a transition to chronic inflammation and should be investigated in more detail. Since the melanoma models remained viable for a minimum of 12 days without signs of biological deterioration and share a significant proportion of prognostic markers derived from human melanoma biopsies (Gerami et al., 2015), we claim that they represent parts of the *in vivo* situation and are well-suited to further investigate the causal relationship between skin bacteria and melanoma. In this proof-of-concept study, skin bacteria from non-melanoma sites were used and the effects are specific to the single healthy donor, which constitutes a limitation. Further studies should incorporate multiple donors to generalize the results to a wider population. Moreover, the impact of skin microbiota from melanoma sites as opposed to non-melanoma sites should be characterized. In addition to melanoma models, the inoculation of non-melanoma models with the same inoculum may offer additional understanding of whether the observed changes in biological responses are primarily influenced by cultivation conditions or reflect specific host–microbe interactions. To achieve higher reproducibility mono-colonization with melanoma-related bacterial species or colonization together with a defined bacterial community, may provide valuable insights into the causal relationship of the skin microbiome in melanoma. However, to fully exploit the complex immune–tumor–microbe crosstalk, the integration of immune cells, such as macrophages or dendritic cells, is required.

Our approach demonstrates that *in situ* melanoma models are appropriate to investigate host–microbe interactions to understand the impact of bacterial colonization in melanoma. This is significant for future research and will increase our understanding of the skin microbiome’s role in melanoma biology and may guide future microbiome-targeted therapeutic strategies.

## Data Availability

The raw data of the transcriptome files have been deposited in NCBI Gene Expression Omnibus (GEO) and are accessible through GEO Series accession number GSE273669. The 16S rRNA gene amplicon sequencing raw data are available at European Nucleotide Archive (ENA) under the project accession number PRJEB89770.

## Supplementary material

Supplementary Figure S1 Principal component analysis (PCA) plot of normalized transcriptome data from epidermis samples of colonized and non-colonized control melanoma models. Colored dots refer to condition and time point of the models. Percentage of variation accounted for by each principal component is shown in brackets by the axis label.

Supplementary Figure S2 Gene ontology enrichment analysis for biological processes in colonized melanoma models on day 12. Dot-plot based on significantly downregulated genes showing induced pathways in colonized models compared to non-colonized control (*n =* 3 biological replicates for each condition and time point; except from non-colonized control melanoma models on day 4 with *n =* 2 biological replicates).

Supplementary Figure S3 Cytokine release of colonized melanoma models normalized to non-colonized control melanoma models over the cultivation period of 12 days. Various cytokines were detected in the cell culture supernatant and measured using flow cytometry-based multiplex immunoassay. Data are presented as mean with ± SD of fold changes relative to non-colonized control melanoma models, with a subtraction of one, from *n =* 4, biological replicates for each time point and condition. Differences between non-colonized control and colonized melanoma models were analyzed by a multiple unpaired t-test with correction for multiple comparison using the Holm–Šidák method (***p <* 0.01, ****p <* 0.01).
