# Supplementary figures and images for: Bacterial colonized melanoma skin models allow to study host–microbe interactions in situ
Source: Front Microbiol. 2026 Feb 24;17:1736700. doi: 10.3389/fmicb.2026.1736700 (PMC12973510; doi:10.3389/fmicb.2026.1736700)

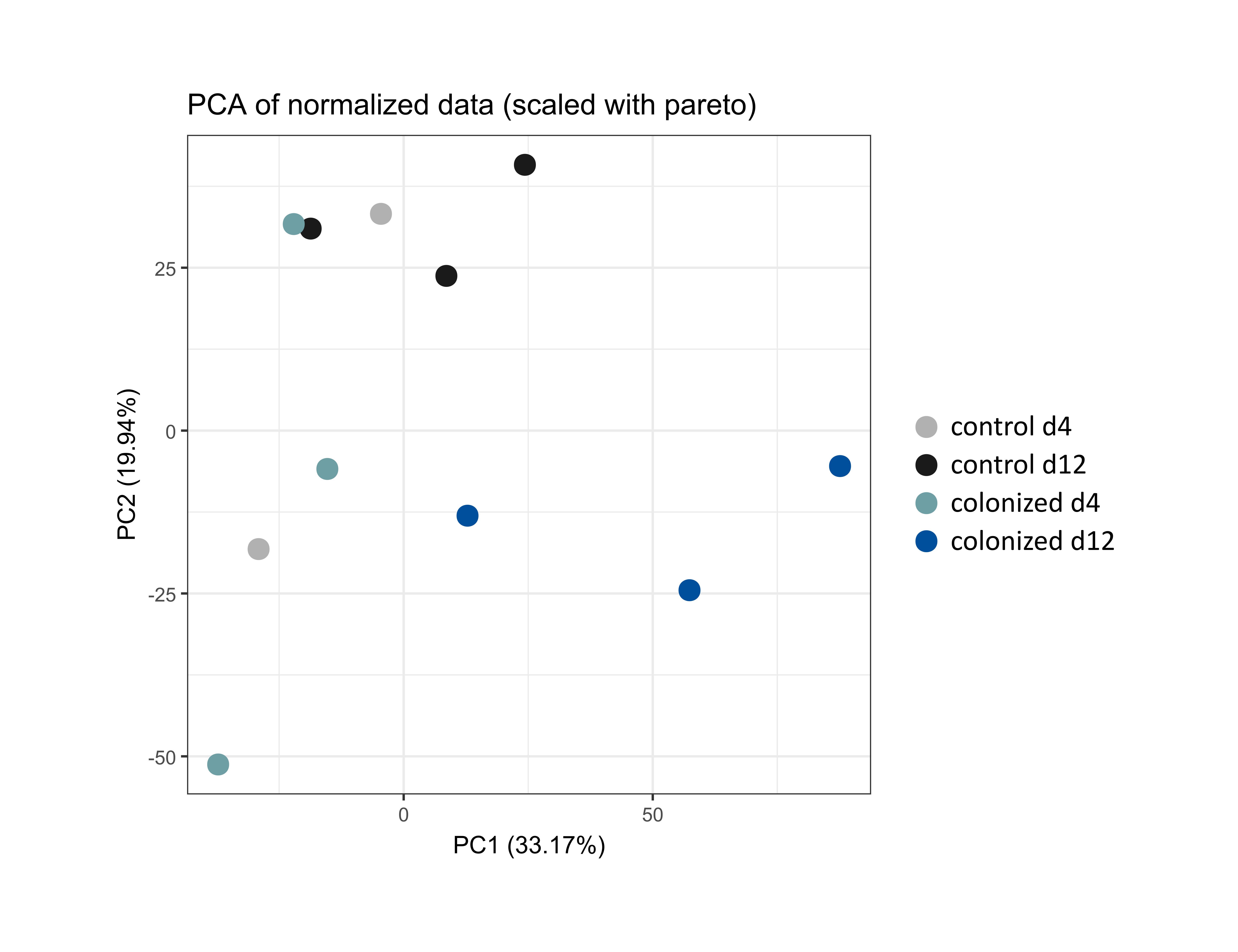

Supplement: Supplementary Figure S1 — Principal component analysis (PCA) plot of normalized transcriptome data from epidermis samples of colonized and non-colonized control melanoma models. Colored dots refer to condition and time point of the models. Percentage of variation accounted for by each principal component is shown in brackets by the axis label. [file Image_1.JPEG]

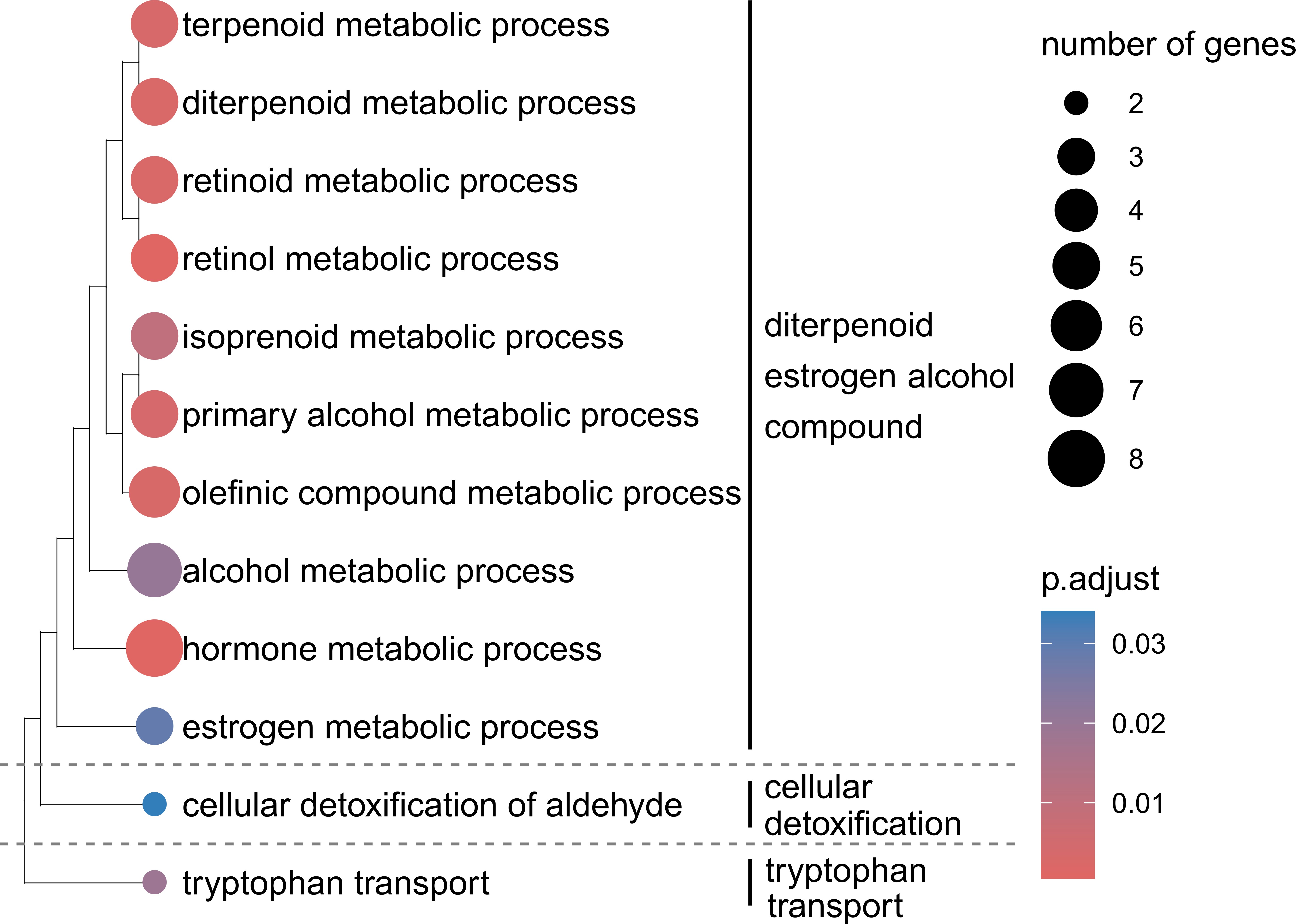

Supplement: Supplementary Figure S2 — Gene ontology enrichment analysis for biological processes in colonized melanoma models on day 12. Dot-plot based on significantly downregulated genes showing induced pathways in colonized models compared to non-colonized control (n = 3 biological replicates for each condition and time point; except from non-colonized control melanoma models on day 4 with n = 2 biological replicates). [file Image_2.JPEG]

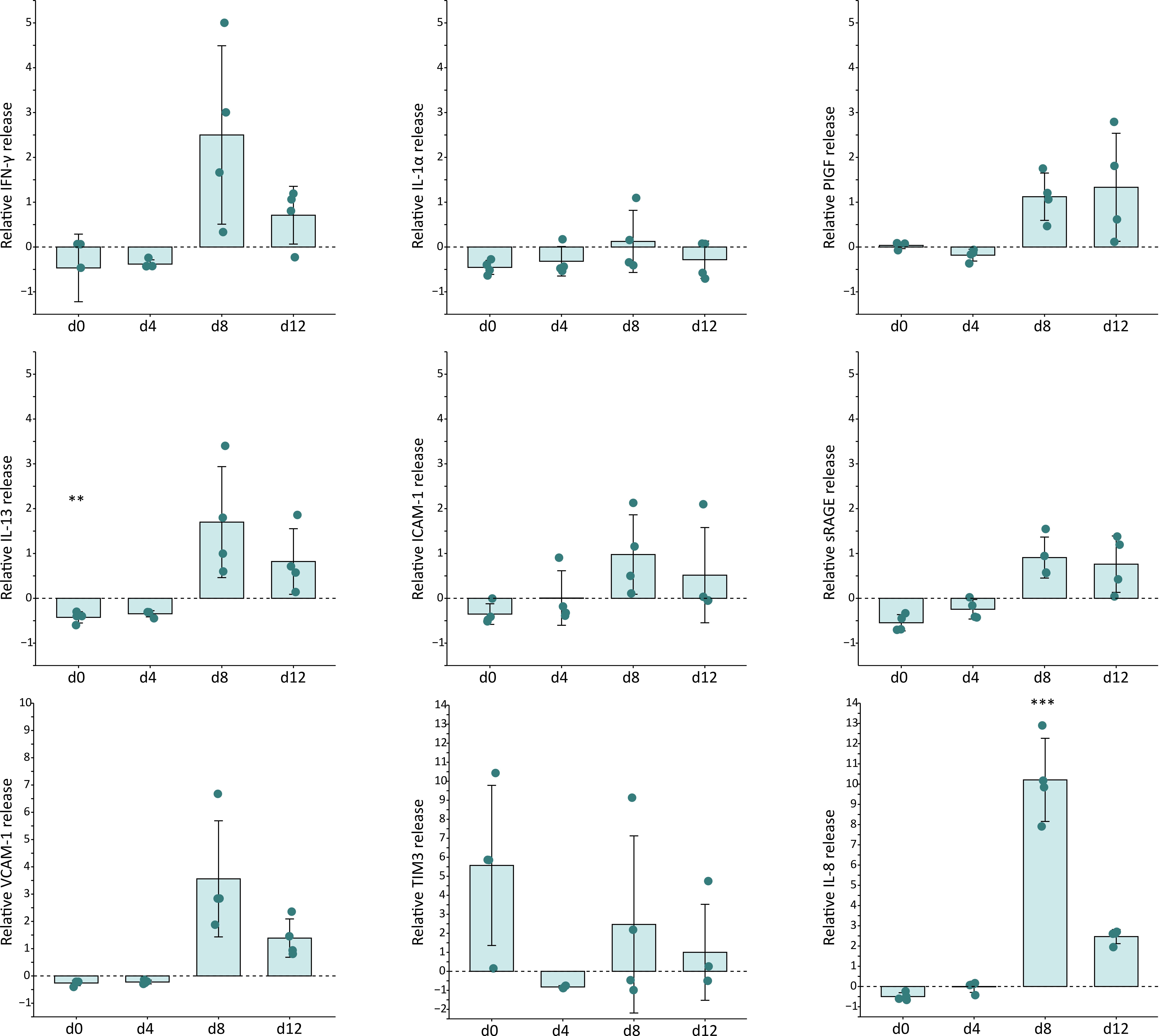

Supplement: Supplementary Figure S3 — Cytokine release of colonized melanoma models normalized to non-colonized control melanoma models over the cultivation period of 12 days. Various cytokines were detected in the cell culture supernatant and measured using flow cytometry-based multiplex immunoassay. Data are presented as mean with ± SD of fold changes relative to non-colonized control melanoma models, with a subtraction of one, from n = 4, biological replicates for each time point and condition. Differences between non-colonized control and colonized melanoma models were analyzed by a multiple unpaired t-test with correction for multiple comparison using the Holm–Šidák method (**p < 0.01, ***p < 0.01). [file Image_3.JPEG]
